# Targeting G _i/o_ -coupled GPCRs to inhibit nociceptors: insights from the serotonin receptor Htr1b and triptans

**DOI:** 10.64898/2026.04.22.719367

**Published:** 2026-06-17

**Authors:** Jing Peng, Brianna T. Sanchez, Anda M. Chirila, Xiangsunze Zeng, Michelle M. DeLisle, Lijun Qi, Jiayin Xiao, Karina Lezgiyeva, Sarah A. Low, Clifford J. Woolf, Nikhil Sharma, David D. Ginty

## Abstract

Pain perception is initiated upon activation of nociceptors of the dorsal root ganglia (DRG) and trigeminal ganglia. We identified G protein-coupled receptors (GPCRs) expressed in CGRP ^+^ mouse and human DRG neurons and found that agonists of several identified G _i/o_ -coupled and orphan GPCRs attenuated neuronal excitability. Experiments focusing on the G _i/o_ -coupled serotonin receptor Htr1b, which is expressed in mouse and human CGRP ^+^ DRG neurons, revealed that Htr1b/1d agonists, the triptans sumatriptan and zolmitriptan, attenuated CGRP ^+^ neuron excitability *in vitro* and exhibited analgesia across several pain models, including neuropathic pain. Conditional genetic deletion experiments showed that triptan-induced analgesia is mediated by Htr1b expressed in A-fiber mechanonociceptors. Also, triptan-associated adverse effects are partially mediated by Htr1b-independent targets. Further testing identified the GPCR Gpr19 as an additional promising target for treating pain. These findings establish a preclinical screening platform for identifying novel analgesics and reveal nociceptor GPCRs that may be targeted to treat pain.

## Full Text Availability

The license terms selected by the author(s) for this preprint version do not permit archiving in PMC. The full text is available from the preprint server.

